# GnRH Agonist Trigger and LH Activity Luteal Phase Support versus hCG Trigger and Conventional Luteal Phase Support in Fresh Embryo Transfer IVF/ICSI Cycles—A Systematic PRISMA Review and Meta-analysis

**DOI:** 10.3389/fendo.2017.00116

**Published:** 2017-06-07

**Authors:** Thor Haahr, Matheus Roque, Sandro C. Esteves, Peter Humaidan

**Affiliations:** ^1^The Fertility Clinic Skive Regional Hospital, Skive, Denmark; ^2^Faculty of Health, Aarhus University, Aarhus C, Denmark; ^3^ORIGEN – Center for Reproductive Medicine, Rio de Janeiro, Brazil; ^4^ANDROFERT, Andrology and Human Reproduction Clinic, São Paulo, Brazil; ^5^Department of Surgery, University of Campinas (UNICAMP), São Paulo, Brazil

**Keywords:** *in vitro* fertilization, intracytoplasmic sperm injection, ovarian stimulation, ovulation induction, gonadotropin-releasing hormone agonist trigger, luteal phase support, live birth rate, ovarian hyperstimulation syndrome

## Abstract

**Introduction:**

The use of GnRH agonist (GnRHa) for final oocyte maturation trigger in oocyte donation and elective frozen embryo transfer cycles is well established due to lower ovarian hyperstimulation syndrome (OHSS) rates as compared to hCG trigger. A recent Cochrane meta-analysis concluded that GnRHa trigger was associated with reduced live birth rates (LBRs) in fresh autologous IVF cycles compared to hCG trigger. However, the evidence is not unequivocal, and recent trials have found encouraging reproductive outcomes among couples undergoing GnRHa trigger and individualized luteal LH activity support. Thus, the aim was to compare GnRHa trigger followed by luteal LH activity support with hCG trigger in IVF patients undergoing fresh embryo transfer.

**Material and methods:**

We conducted a systematic review and meta-analysis of randomized trials published until December 14, 2016. The population was infertile patients submitted to IVF/ICSI cycles with GnRH antagonist cotreatment who underwent fresh embryo transfer. The intervention was GnRHa trigger followed by LH activity luteal phase support (LPS). The comparator was hCG trigger followed by a standard LPS. The critical outcome measures were LBR and OHSS rate. The secondary outcome measures were number of oocytes retrieved, clinical and ongoing pregnancy rates, and miscarriage rates.

**Results:**

A total of five studies met the selection criteria comprising a total of 859 patients. The LBR was not significantly different between the GnRHa and hCG trigger groups (OR 0.84, 95% CI 0.62, 1.14). OHSS was reported in a total of 4/413 cases in the GnRHa group compared to 7/413 in the hCG group (OR 0.48, 95% CI 0.15, 1.60). We observed a slight, but non-significant increase in miscarriage rate in the GnRHa triggered group compared to the hCG group (OR 1.85; 95% CI 0.97, 3.54).

**Conclusion:**

GnRHa trigger with LH activity LPS resulted in comparable LBRs compared to hCG trigger. The most recent trials reported LBRs close to unity indicating that individualization of the LH activity LPS improved the luteal phase deficiency reported in the first GnRHa trigger studies. However, LPS optimization is needed to further limit OHSS in the subgroup of normoresponder patients (<14 follicles ≥ 11 mm).

**Prospero registration number:**

CRD42016051091

## Introduction

GnRH agonist (GnRHa) trigger for final oocyte maturation significantly reduces the risk of ovarian hyperstimulation syndrome (OHSS) in both fresh transfer and segmentation IVF/ICSI cycles (1, 2). Despite being well established as first-line treatment in oocyte donation and segmentation cycles, the use of GnRHa trigger for fresh embryo transfer cycles remains subject to debate due to equivocal results concerning the reproductive outcomes (2). Importantly, the lower reproductive outcomes seen during the first randomized controlled trial (RCT) using GnRHa trigger were caused by a severe luteal phase deficiency that was not overcome by a standard luteal phase support (LPS) (3, 4). The pathophysiological mechanism behind the luteal phase insufficiency was low-circulating endogenous LH levels after the GnRHa trigger, leading to corpus luteum demise and consequently suboptimal progesterone levels at peri-implantation (5–7). Consequently, this finding led to the development of a modified LPS, which has proven to be mandatory to obtain reproductive outcomes comparable to those seen after hCG trigger (5). During the last decade, two different modified LPS strategies have been proposed to overcome the aforementioned luteal phase deficiency (7). One of these approaches has been called the “European approach” in which the endogenous steroid (progesterone and estradiol) production by the corpora lutea is boosted by exogenous LH activity, i.e., LH or hCG after GnRHa trigger. The other approach has been called the “American approach” in which luteal progesterone and estradiol are administered exogenously, thus, disregarding the function of the corpora lutea (7).

Despite the efforts to optimize the LPS after GnRHa trigger, a recent Cochrane review and meta-analysis concluded that GnRHa as a final oocyte maturation trigger in fresh autologous cycles is associated with a lower live birth rate (LBR), a lower ongoing pregnancy rate (pregnancy beyond 12 weeks), and a higher early miscarriage rate (less than 12 weeks) compared to hCG trigger (2). In the Cochrane review, it was suggested that the use of a GnRHa trigger would be useful, only, in cases in which a freeze-all strategy (segmentation) would be implemented (for whatever reason), for oocyte donor cycles and in fertility preservation patients (2).

As Cochrane reviews are internationally recognized standards for evidence-based health care, many fertility specialists subsequently have opted to segment the cycle whenever a GnRHa trigger is applied. However, the conclusion that GnRHa trigger should not be used in fresh transfer cycles was subsequently criticized due to the pooled analyses of heterogeneous studies, using different LPS policies, or no LPS at all after GnRHa trigger (8).

Since the clinical decision to change to GnRHa trigger instead of hCG trigger in fresh transfer GnRH antagonist cycles has been a matter of debate, and due to the clinical implications of such an intervention, the role of GnRHa trigger followed by LH activity luteal phase support (LPS) needs to be re-examined in view of the new evidence that has emerged after the publication of the latest Cochrane review. We, therefore, conducted a systematic review to aggregate the existing data on the effect of GnRHa trigger and LPS in fresh embryo transfer IVF/ICSI cycles, using meta-analysis as a method. The aims were to summarize the evidence for each outcome investigated and rate its strength based on GRADE.

## Materials and Methods

We adhered to the Preferred Reporting Items for Systematic Reviews and Meta-Analyses (PRISMA) guidelines (9) and added the PRISMA checklist in the Supplementary Material. The study protocol is accessible at http://www.crd.york.ac.uk/PROSPERO/ with registration number CRD42016051091.

### Search Strategy

The literature search was performed in MEDLINE/PubMed and Embase databases from 1974 to December 14, 2016, with the assistance of a trained research librarian. The search strategy is provided as Supplementary Material. We limited the search to RCTs published in English, comparing hCG versus GnRHa trigger in infertile patients, undergoing controlled ovarian stimulation for IVF or ICSI in GnRH antagonist co-treated cycles. Cross-over trials and conference abstracts were not considered.

### Selection of Studies and Validity Assessment

The selection criteria are described according to PICO (Patients, Intervention, Comparison, and Outcomes) questions, provided in Table S1 in Supplementary Material. Articles were only included if they investigated LPS, which specifically means, luteal supplementation with LH activity in the form of either exogenous hCG or LH to boost the endogenous steroid production. The reason stems from the overwhelming evidence against conventional LPS in GnRHa triggered fresh embryo transfer IVF/ICSI cycles (2, 3, 7). Furthermore, we did not include dual trigger studies because this concept is physiologically different from GnRHa trigger and LPS (10). Oocyte donation and frozen-thawed embryo transfer cycles were also excluded. Citations were managed in Covidence© (Vertitas Health Innovation Ltd.). Duplicates were removed, and subsequently all citations were screened by title and abstract by two independent reviewers (Thor Haahr and Matheus Roque). Any discrepancies were solved by agreement, and if needed they reached consensus with the senior authors (Sandro C. Esteves and Peter Humaidan). Thereafter, the full texts of eligible RCTs were obtained. The authors evaluated the studies’ eligibility and subsequently extracted the data after the Cochrane risk of bias tool was used to assess the risk of bias in the included studies.

### Data Extraction

Data was extracted in summary of finding (SOF) tables both individually for each study and also compiled for each outcome (Supplementary SOF tables). The critical outcome measures were LBR and OHSS rate. The secondary outcome measures were the number of oocytes retrieved, the number of M2 oocytes, the number of high-quality embryos, clinical pregnancy rate, miscarriage rate, and ongoing pregnancy rate. Authors of incomplete datasets were contacted to request that they provided live birth data for this meta-analysis (5, 11). Unfortunately, we could not extract relevant data on M2 oocytes from the included studies.

The GRADE quality of evidence was used to determine the strength of evidence for each outcome according to the GRADE handbook (12).

With only minor changes, the definitions of outcomes adhered to the ICMART/WHO glossary (13). LBR was defined as the ratio between the number of deliveries resulting in at least one live birth and the number of cycles randomized (i.e., intention to treat). OHSS was defined according to recently established criteria, which stated that: (i) the subject should had undergone ovarian stimulation (OS) AND had received a trigger dose for final oocyte maturation (e.g., hCG, GnRHa, or kisspeptin) followed by either fresh transfer or segmentation (cryopreservation of embryos) or (ii) the subject had undergone OS AND had a positive pregnancy test (14). Clinical pregnancy was defined as a pregnancy diagnosed by ultrasonographic visualization of one or more gestational sacs. Ongoing pregnancy was defined as a viable pregnancy at 11 weeks’ gestation (6). Miscarriage rate was defined as the loss of a clinical pregnancy at any gestational age before live birth. Good quality embryos and the number of M2 oocytes were assessed according to what was reported in the publications.

### Quantitative Analysis

Forest plots were computed in Review Manager, Version 5.3, Copenhagen: The Nordic Cochrane Centre, The Cochrane Collaboration, 2014. All plots were made per intention to treat, defined as including all randomized participants in the denominator except for missing patients who were excluded. Heterogeneity was evaluated with *I*-squared statistic (*I*^2^). Statistical significance was set at a *p*-value < 0.05. A fixed effects model was chosen if heterogeneity (*I*^2^) among studies was below 50%. If the *I^2^* was higher than 50%, the heterogeneity was considered substantial, and the random-effects model was applied. Dichotomous outcomes were reported as odds ratios, using a Mantel–Haenszel method, and continuous outcomes as mean differences with the use of inverse variance method. Sensitivity analyses and assessment of publication bias were conducted to assess the influence of individual studies on the results (Supplementary Material). Sensitivity analysis was performed for all outcomes. Finally, we performed a subgroup analysis among patients receiving LPS after GnRHa trigger. In particular, we compared LPS given as a single bolus of hCG at 36-h post-trigger versus individualized LPS, i.e., when additional boluses of hCG were given either at oocyte pickup (OPU) plus 5 days or daily. We extracted this information from the studies of Humaidan et al. (5) and Humaidan et al. (11). However, the patients receiving LPS 12-h posttrigger in the study by Humaidan et al. (5) were disregarded as it has been shown that this strategy leads to a markedly poor reproductive outcome (5).

## Results

A total of 694 unique citations were identified and subjected to initial screening of titles and abstracts. Subsequently, a total of 31 citations were eligible for full-text reading. Among these, five studies met the selection criteria and were scrutinized for qualitative and quantitative analysis (5, 6, 11, 15, 16). The full selection process is depicted in the PRISMA flowchart, Figure S1 in Supplementary Material. The characteristics of included studies are provided for each individual study in Tables S2–S6 in Supplementary Material. Furthermore, a summary of findings is provided for overall outcome assessment in Table 1.

**Table 1:** **Summary of findings table, GnRH agonist (GnRHa) trigger compared to hCG trigger for final oocyte maturation**.

Quality assessment							No. of patients		Effect		Quality	Importance
No. of studies	Study design	Risk of bias	Inconsistency	Indirectness	Imprecision	Other considerations	GnRHa	hCG	Relative (95% CI)	Absolute (95% CI)		
**Live birth**												
5	Randomized trials	Serious^a^	Not serious	Not serious	Serious^b^	None	116/444 (26.1%)	119/413 (28.8%)	OR 0.84 (0.62–1.14)	34 fewer per 1,000 (from 28 more to 88 fewer)	⊕⊕⊖⊖ LOW	Critical
**Ovarian hyperstimulation syndrome (OHSS)**												
5	Randomized trials	Serious^c^	Not serious	Serious^d^	Serious	None	4/446 (0.9%)	7/413 (1.7%)	OR 0.48 (0.15–1.60)	10 fewer per 1,000 (from 30 fewer to 10 more)	⊕⊖⊖⊖ VERY LOW	Critical
**Ongoing pregnancy rate**												
2	Randomized trials	Serious^c^	Serious^e^	Not serious	Serious^b^	None	94/337 (27.9%)	100/349 (28.7%)	OR 0.95 (0.59–1.53)	10 fewer per 1,000 (from 94 more to 95 fewer)	⊕⊖⊖⊖ VERY LOW	Important
**Clinical pregnancy rate**												
5	Randomized trials	Serious^c^	Not serious	Not serious	Serious^b^	None	147/446 (33.0%)	136/413 (32.9%)	OR 0.99 (0.74–1.32)	2 fewer per 1,000 (from 63 fewer to 64 more)	⊕⊕⊖⊖ LOW	Important
**Miscarriage**												
5	Randomized trials	Serious^a^	Not serious	Not serious	Serious^b^	None	29/145 (20.0%)	17/136 (12.5%)	OR 1.85 (0.97 to 3.54)	84 more per 1,000 (from 3 fewer to 211 more)	⊕⊕⊖⊖ LOW	Important
**Oocytes aspirated**												
4	Randomized trials	Serious^c^	Serious^e^	Not serious	Serious^b^	None	261	214	–	MD 0.25 higher (2.03 lower to 2.53 higher)	⊕⊖⊖⊖ VERY LOW	Important
**Good quality embryos**												
2	Randomized trials	Serious^c^	Not serious	Not serious	Serious^b^	None	79	49	–	MD 0.94 higher (0.01 higher to 1.87 higher)	⊕⊕⊖⊖ LOW	Important

*Population: patients submitted to IVF/ICSI cycles in GnRH antagonist protocol with fresh embryo transfer*.

*Intervention: GnRHa trigger followed by a modified luteal phase support (LPS) with LH activity*.

*Comparison: hCG with standard LPS*.

*CI, confidence interval; OR, odds ratio; MD, mean difference*.

*^a^Regarding risk of bias, serious limitations as most studies were underpowered and open label. Furthermore, data from Ref. (5, 11) were extracted from unpublished data*.

*^b^The risk of imprecision was high as the 95% CI did include figures which might lead to other conclusions*.

*^c^Regarding risk of bias, serious limitations as most studies were underpowered and open label*.

*^d^The indirectness was moderate as both high-risk and low-risk OHSS patient groups were compiled. Furthermore, GnRHa trigger agent and modified LPS was not uniform and there could have been different OHSS definitions*.

*^e^I^2^ heterogeneity above 50%*.

*⊕⊖⊖⊖, very low evidence; we have very little confidence in the effect estimate: the true effect is likely to be substantially different from the estimate of effect*.

*⊕⊕⊖⊖, low evidence; our confidence in the effect estimate is limited: the true effect may be substantially different from the estimate of the effect*.

### Live Birth Rate

**Figure 1:**
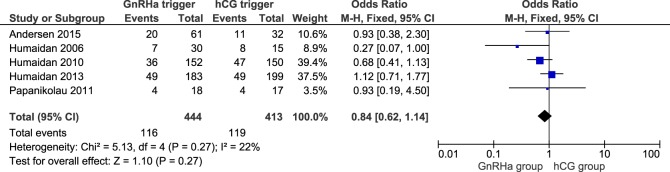
**GnRH agonist (GnRHa) trigger + modified luteal phase support with LH activity versus hCG trigger, critical outcome live birth/intention to treat**. Two patients were missing in Ref. (11).

**Figure 2:**
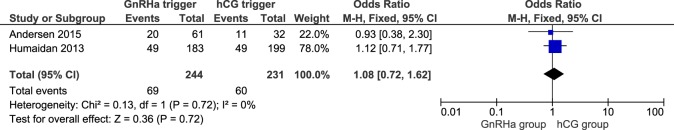
**Subgroup analysis—including only the two most recent studies**.

Live birth data were obtained from all included trials with a total of 857 cycles included. LBRs in the GnRHa and hCG groups were 26.1 and 28.8%, respectively. The corresponding OR for LBR was 0.84 (95% CI 0.62, 1.14, *I^2^* = 22%; Figure 1). According to GRADE, the quality of evidence was low (Table 1). Further, subgroup analysis indicated that LBR was very close to unity in the most recent publications which introduced individualized LPS (11, 16), with an OR 1.08 (95% CI 0.72, 1.62, *I^2^* = 0%; Figure 2). In order to evaluate if there was any study that could influence the conclusions regarding LBR, we performed a sensitivity analysis removing study by study. In general, the observed pooled effect estimate was not significantly affected by the removal of any of the studies, indicating that LBR was not statistically different between the groups, independent of the evaluated scenario. The lowest and highest OR were, respectively: OR 0.68 (95% CI 0.45, 1.01, *I^2^* = 0%, Figure S2 in Supplementary Material) when removing (11); and OR 0.95 (95% CI 0.65, 1.38, *I^2^* = 26%; Figure S3 in Supplementary Material) removing (6). Finally, in a subgroup analysis considering LPS with hCG bolus at OPU only, the pooled OR was 0.78 (95% CI 0.52, 1.18, *I^2^* = 0%; Figure S4 in Supplementary Material).

### OHSS Rate

**Figure 3:**
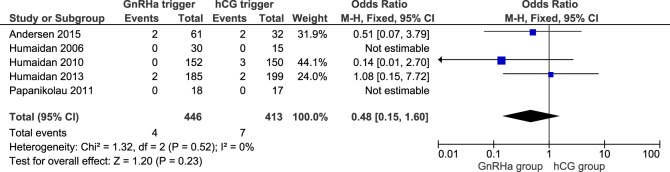
**GnRH agonist (GnRHa) trigger + modified luteal phase support with LH activity versus hCG trigger, critical outcome ovarian hyperstimulation syndrome/intention to treat**.

All studies reported OHSS rates and all OHSS cases were moderate late-onset according to criteria established recently (14). The OHSS rates in the GnRHa and hCG groups were 0.9 and 1.7%, respectively, and the corresponding OR was 0.48 (95% CI 0.15, 1.60, *I^2^* = 0%; Figure 3). A lower OHSS rate was observed in the GnRHa trigger group although not statistically significant. In the corresponding subanalysis to Figure S4 in Supplementary Material, investigating only the design in which hCG was administered at OPU (36 h post-trigger), no OHSS case was observed (figure not shown). Sensitivity analyses showed that the observed pooled effect size was not significantly affected by the removal of any study (Table S8 in Supplementary Material). According to GRADE, the quality of evidence was very low (Table 1).

### Ongoing Pregnancy and Clinical Pregnancy

**Figure 4:**
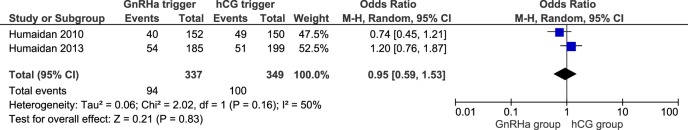
**GnRH agonist (GnRHa) trigger + modified luteal phase support with LH activity versus hCG trigger, important outcome Ongoing pregnancy/intention to treat**.

**Figure 5:**
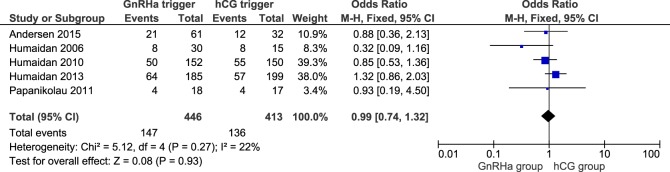
**GnRH agonist (GnRHa) trigger + modified luteal phase support with LH activity versus hCG trigger, important outcome Clinical pregnancy/intention to treat**.

Two studies reported ongoing pregnancy rates, including 686 cycles (6, 11). The ongoing pregnancy rate in the GnRHa and hCG groups were 27.9 and 28.7%, respectively. No significant difference was observed in ongoing pregnancy with an effect estimate close to unity (OR 0.95, 95% CI 0.59, 1.53; *I^2^* = 50%; Figure 4). All studies reported the clinical pregnancy rate, including 859 cycles. The clinical pregnancy rate in the GnRHa and hCG groups were 33 and 34%. Overall, pooled results indicated that the clinical pregnancy rate was similar comparing GnRHa + LPS and hCG trigger. The OR was 0.99 (95% CI 0.74, 1.32, *I^2^* = 22%; Figure 5). Sensitivity analyses were performed only for clinical pregnancy rate, as there were only two studies included in ongoing pregnancy rate analysis. Sensitivity analysis for clinical pregnancy rate showed that the observed pooled effect size was not significantly affected by the removal of any study. According to GRADE, the quality of evidence was very low for ongoing pregnancy rate and low for clinical pregnancy rate (Table 1).

### Miscarriage Rate

**Figure 6:**
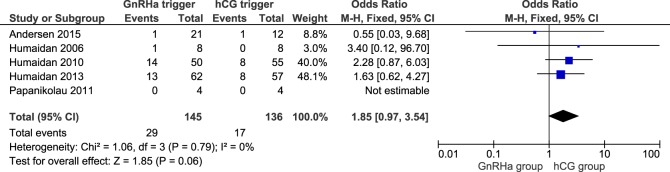
**GnRH agonist (GnRHa) trigger + modified luteal phase support with LH activity versus hCG trigger, important outcome miscarriage/intention to treat**. Two patients were missing in Ref. (11), both in the GnRHa group. If they were both lost pregnancies, then the effect estimate would have been significant: OR 1.97 (1.03–3.75).

In a total of 281 clinical pregnancies, we observed a slight albeit non-significant higher miscarriage rate when comparing the GnRHa triggered group to the hCG group (OR 1.85; 95% CI 0.97, 3.54; *I^2^* = 0%; Figure 6). The miscarriage rate was 20.0% in GnRHa group and 12.5% in hCG group, respectively (*p* = 0.06). According to GRADE, the level of evidence was low (Table 1). Sensitivity analysis demonstrated that the study of Andersen et al. had an influence on the results; when this study was removed, the clinical pregnancy loss was significantly increased in the GnRHa triggered group (OR: 1.97, 95% CI 1.01, 3.85; *I^2^* = 0%). The removal of any other study did not impact the results substantially.

### Oocytes Retrieved, M2 Oocytes, and Good Quality Embryos

**Figure 7:**
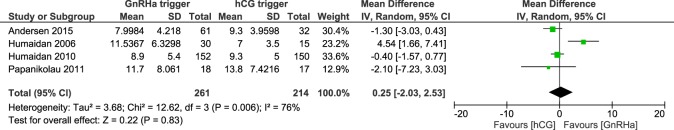
**GnRH agonist (GnRHa) trigger versus hCG trigger, important outcome Oocytes retrieved/intention to treat**.

**Figure 8:**
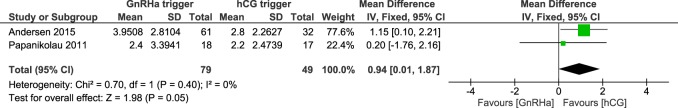
**GnRH agonist (GnRHa) trigger versus hCG trigger, important outcome good quality embryos/intention to treat**. Good quality embryos defined differently. In Ref. (16) as Grade 0, 1 day 2 or 3. In Ref. (15), a composite mean of embryos transferred and embryos frozen.

We were able to extract data from four studies on the number of oocytes retrieved (5, 6, 15, 16). The mean (SD) number of oocytes retrieved was 8.0 (4.2) and 9.3 (3.9) in the GnRHa and hCG groups, respectively. Overall, the results indicated that the number of oocytes retrieved was not different between groups (OR 0.98, 95% CI 0.73, 1.30; *I^2^* = 76%; Figure 7). The mean difference in the number of oocytes retrieved was 0.25 (95% CI −2.03, 2.53). Due to the high heterogeneity, we applied the random effect model to perform the analysis. Two studies reported data on the number of good quality embryos (15, 16). A significant difference was observed in favor of GnRHa trigger regarding the number of good quality embryos (MD 0.94, 95% CI 0.01, 1.87; Figure 8). However, according to GRADE, the level of evidence was low (Table 1).

## Discussion

### Summary of Main Results

With conflicting evidence concerning the use of GnRHa trigger in fresh transfer IVF/ICSI cycles and the inherent risk and clinical implications associated with this intervention, we felt a need to clarify the role of the modified LPS with LH activity in this particular patient population. To our knowledge, this is the first PRISMA systematic review and meta-analysis summarizing the evidence currently available concerning GnRHa trigger followed by LPS in patients undergoing IVF/ICSI and fresh embryo transfer, including new evidence published after the latest Cochrane review (2). In our analysis, there was a slightly lower but non-significant difference in LBR in the GnRHa group compared to the hCG group. Importantly, in the newer studies employing individualized LPS, the odds ratio was close to unity regarding LBR. Furthermore, a lower OHSS rate was observed in the GnRHa trigger group compared to the hCG trigger group, although not statistically significant. However, the use of GnRHa trigger with LPS was associated with increased miscarriage rates in a sensitivity analysis and also the absolute effect estimates given in Table 1, i.e. 84 more miscarriages (95% CI from 3 fewer to 211 more) in GnRHa triggered cycles would suggest that although LBR was comparable to hCG triggered cycles, a further optimization of LBR might be achieved. As for the number of oocytes retrieved, the use of GnRHa trigger in preference over hCG trigger resulted in no apparent difference. However, the number of good quality embryos was significantly higher in the GnRHa group.

The strength of evidence for all outcomes was low or very low according to GRADE, which means that although no significant differences were observed, we are overall uncertain about the effect estimates. It is therefore very likely that further research will have a substantial impact on the observed effect estimates.

### Interpretation of Results and Clinical Considerations

In the waiting time for future research that could further clarify the abovementioned effect estimates, clinical decision-making should also take into account the clinical and biological plausibility as well as the standpoint of the individual patient (17, 18). In the discussion below, we scrutinize the forest plots with the aim to assist clinicians to choose the optimal trigger strategy, GnRHa or hCG and the subsequent LPS needed in fresh transfer GnRH antagonist cotreated cycles.

First, although LBR after fresh embryo transfer was comparable between GnRHa and hCG triggered cycles, there was an overall trend toward lower LBR with GnRHa. This effect was mainly due to the higher miscarriage rates observed with the use of GnRHa trigger. However, miscarriage rates were reduced in the latest studies that provided additional boluses of hCG during LPS, the so-called individualized LPS (11, 16), resulting in a pooled OR concerning LBR close to unity when GnRHa and hCG trigger were compared (11, 16). Such disparity between the older and more recent RCTs indicate heterogeneity even among the highly selected RCTs included in the present study. One major issue in this aspect is the fact that the concept of GnRHa trigger and LPS has mostly been developed through pilot trials which were underpowered to adequately investigate superiority of the experimental arms of LPS (5, 11, 15, 16). The aggregation of such experimental arms in meta-analyses would increase the power, albeit the need for caution in the interpretations would also increase due to heterogeneity, in this case especially in the LPS. To give an example, in Ref. (5), it was clearly shown that hCG administration (1,500 IU) at 12 h post-trigger resulted in only 2/17 (11.8%) clinical pregnancies, including an additional early pregnancy loss (5). Erroneously, such an experimental design that was proven ineffective had a negative impact on the overall pooled OR of LBR in Figure 1. In another interesting proof of concept study by Papanikolaou et al., LPS was given in the form of LH injections every other day from the OPU onward for 10 days (15). The authors reported comparable LBR and no OHSS in either group; however, this approach has not been corroborated in larger trials. Furthermore, in the study by Humaidan et al. (11), two moderate OHSS cases were recorded in the low-risk OHSS population who received an additional hCG bolus at OPU + 5. This finding prompted the authors to state that further refinement of the additional hCG bolus was needed for this population (11).

A refinement in modified LPS is the addition of a daily microdose of hCG (125 IU) from OPU until the day of the pregnancy test, as proposed by Andersen and colleagues in their proof of concept trial (16). In this study, the authors eliminated any form of standard LPS in an attempt to explore the exogenous free LPS after GnRHa trigger, initially proposed by Kol et al. (19, 20). Interestingly, in the study by Andersen et al., the miscarriage rate was lower albeit non-significant in the GnRHa trigger group compared to hCG trigger. Moreover, a recent retrospective analysis reported a lower pregnancy loss and a 9% higher clinical pregnancy rate (LBR not reported) when 100 IU/L hCG daily was compared to the bolus of 1,500 hCG at OPU + 5 (21). In a subgroup analysis, the individualized LPS approach resulted in a pooled effect estimate of LBR close to unity and slightly in favor of GnRHa trigger, Figure 2. Based on these results, we conclude that in subpopulations such as normoresponder patients (<14 follicles ≥ 11 mm) (22), there is a need for additional LPS after GnRHa trigger to achieve comparable reproductive outcomes compared to hCG trigger.

Second, our subgroup analysis pooling the studies of LPS given as a single bolus of hCG 36 h post-GnRHa trigger revealed no OHSS cases, with a LBR non-inferior to that of hCG trigger (Supplementary Material; Figure 4). Hence, taking also the evidence from cohort studies into account (23), administering a single bolus of hCG at OPU as a means of LPS would limit OHSS to a minimum. Moreover, pooled data from the studies that utilized individualized LPS after GnRHa trigger (Figure 2) suggested that additional LPS was beneficial with regard to LBR. Nonetheless, the addition of additional hCG in the luteal phase could increase the risk of OHSS, as indicated by the reported 2.2% (4/186) OHSS cases among patients receiving the individualized regimen compared to none in the LPS with a single bolus of hCG given 36 h post-GnRHa trigger (11, 16). Furthermore, significantly lower OHSS rates have been reported with GnRHa trigger compared with hCG trigger in RCTs and observational studies (24–26); however, these papers were not included in the present meta-analysis as they did not meet the inclusion criteria.

Taken together, it is plausible to conclude that LPS after GnRHa trigger should be individualized according to the number of follicles ≥11 mm on the aspiration day. As an example, normoresponder patients (<14 follicles ≥ 11 mm), who clearly have a lower number of functioning corpora lutea to “boost” than patients with ≥ 14 follicles, would be eligible to receive the individualized LPS. In contrast, hyper-responder patients, who commonly exhibit excessive corpora lutea after trigger, could be given a single bolus of hCG at OPU when a fresh transfer is planned. Consequently, future studies should focus on further fine-tuning of the individualized LPS to secure high LBR and additional reduction in the OHSS rate.

### Limitations and Strengths

Like all meta-analyses, there are limitations that should be taken into consideration. Apart from the previously discussed heterogeneity of the included studies, the number of included studies as well as the sample size was relatively low. Also, bias might have been introduced as data not published as full-text articles and in languages other than English were excluded from our meta-analysis. Moreover, the present analysis was restricted to analyze the “European approach” only, i.e., LPS, after GnRHa trigger. As far as the “American approach” is concerned, Babayof et al. investigated a modified LPS by adding additional exogenous progesterone and estradiol during the luteal phase in OHSS risk patients compared to hCG trigger in GnRH antagonist cotreated cycles (24). The findings of Babayof et al. indicate a lower risk of OHSS in the GnRHa trigger group, however, at the expense of a higher miscarriage rate and a very low LBR. In a subsequent RCT, Engmann and colleagues compared GnRHa trigger followed by modified LPS (American approach) to hCG trigger in long GnRHa downregulated patients (25). A significantly lower OHSS rate was reported in the GnRHa group compared to the hCG triggered group (0 versus 31%, respectively). In that trial, Engmann and colleagues attempted to further extend the exogenous LPS, now adding 50 mg I.M. progesterone daily until the clinical pregnancy scan in week 7. In contrast to Babayof et al., this modification resulted in a non-significant difference in ongoing pregnancy rate between GnRHa trigger and hCG (25). These findings were subsequently supported by the results of a retrospective cohort study performed in Asian women (23). However, at this point due to the paucity of RCTs, further investigation is required before firm conclusions can be drawn concerning the American approach for modified LPS after GnRHa trigger.

Despite these limitations, we highlight that we performed sensitivity analyses to evaluate the potential bias that could occur by each study (Supplementary Material). Even after the sensitivity analysis was performed, there was no statistical difference when evaluating the primary outcome, LBR. Furthermore, we obtained live birth data from Ref. (11), which was not originally included in the authors’ paper and only two patients were missing. Additionally, we rated the strength of evidence using GRADE. The overall low or very low strength of evidence, however, add uncertainty to the estimates, thus, emphasizing the need for further research before firm clinical recommendations can be made.

### Future Aspects

In the future, segmentation will undoubtedly play a bigger role than presently, coinciding with the improvement in cryopreservation techniques globally, and thus, the reproductive outcome of frozen-thaw cycles. However, despite the irrefutable OHSS risk reduction after GnRHa trigger followed by segmentation, even in GnRHa triggered segmented cycles, a few severe early-onset OHSS cases have been reported (27–29). Furthermore, the segmentation policy after GnRHa trigger in line with previous reports on health outcomes of children born as a result of cryopreserved thawed embryos is likely to increase the incidence of macrosomia and large for gestational age (30–32), the risk of placenta accreta (33, 34), and the risk of preeclampsia (35). Moreover, this additional elective manipulation of gametes which could induce epigenetic changes might add further to the risk of cardiovascular disorders that have already been reported to be associated with ART (36, 37). Finally, a comprehensive evaluation of elective segmentation taking into account cost-effectiveness, patient-centeredness, and time to live birth has yet to be carried out.

Thus, fresh embryo transfer should not be disregarded and GnRHa trigger can be used to secure both a high LBR and a low OHSS rate. The individualized LPS approach can be introduced to clinical use although a further fine-tuning of the LH activity used during LPS might improve the results even further. Moreover, a cost-effective and patient-centered analysis comparing GnRHa trigger and LPS with the gold standard hCG trigger would allow better judgment of the clinical significance of our findings.

## Conclusion

In fresh transfer cycles triggered with either GnRHa or hCG, LBR is comparable, regardless of the trigger strategy, provided that GnRHa trigger is followed by LPS. Moreover, evidence suggest that individualized LPS could further improve LBR following GnRHa trigger.

## Author Contributions

All authors contributed to study design, manuscript drafting, and critical discussions. TH and MR scrutinized the literature and performed the qualitative and quantitative analysis. All authors contributed to, revised, and accepted the final manuscript.

## Conflict of Interest Statement

PH received unrestricted research grants from MSD, Merck, and Ferring as well as honoraria for lectures from MSD, Merck, and IBSA. SE received honoraria for lectures from Merck and Besins. MR received honoraria for lectures from Merck. TH declares that the research was conducted in the absence of any commercial or financial relationships that could be construed as a potential conflict of interest.
