# Supplementary material for: GnRH Agonist Trigger and LH Activity Luteal Phase Support versus hCG Trigger and Conventional Luteal Phase Support in Fresh Embryo Transfer IVF/ICSI Cycles—A Systematic PRISMA Review and Meta-analysis
Source: Front Endocrinol (Lausanne). 2017 Jun 7;8:116. doi: 10.3389/fendo.2017.00116 (PMC5461358; doi:10.3389/fendo.2017.00116)
Supplement: Supplementary file 1 [file Data_Sheet_1.docx]

**Supplementary material**

Title: GnRH agonist trigger and modified LH activity luteal phase support versus hCG trigger and conventional luteal phase support in fresh embryo transfer IVF/ICSI cycles – a systematic PRISMA review and meta-analysis

Running title: 
GnRHa trigger and modified luteal phase support does not lower the live birth rate

Authors: Thor Haahr^a,b*^, Matheus Roque^c^, Sandro C. Esteves^b,d,e^, Peter Humaidan^a,b^

^a^The Fertility Clinic Skive Regional Hospital, 7800 Skive, Denmark

^b^Faculty of Health, Aarhus University, 8000 Aarhus C, Denmark

^c^ORIGEN – Center for Reproductive Medicine, Rio de Janeiro, RJ, Brazil

^d^ANDROFERT, Andrology and Human Reproduction Clinic, 13075-460 Campinas, SP, Brazil

^e^Department of Surgery, University of Campinas (UNICAMP), Campinas, SP, Brazil

**Contents:**

1. **Table of inclusion criteria (PICO format)**
2. **Individual study characteristics**
3. **Sensitivity analysis**
4. **Publication bias**
5. **Literature search**
6. **PRISMA checklist**
7. **Table of inclusion criteria (PICO format)**

**Supplementary table S1. Selection criteria of the included studies - Population Intervention Comparison Outcome (PICO)**

| **Population** | - Patients submitted to IVF / ICSI cycles in GnRH antagonist protocol with fresh embryo transfer |
| --- | --- |
| **Intervention** | - GnRH agonist trigger followed by a modified luteal phase support with LH activity |
| **Comparison** | - hCG with standard luteal phase support |
| **Outcome** | - Live birth rate - Ongoing pregnancy rate - Clinical pregnancy rate - OHSS - Miscarriage rate - Number of good quality embryos - Number of M2 oocytes - Number of retrieved oocytes |
| **Study type** | - Randomized controlled trials |

1. **Flowchart of inclusion and individual study characteristics**

**Figure S1: Flow chart of included studies**

**
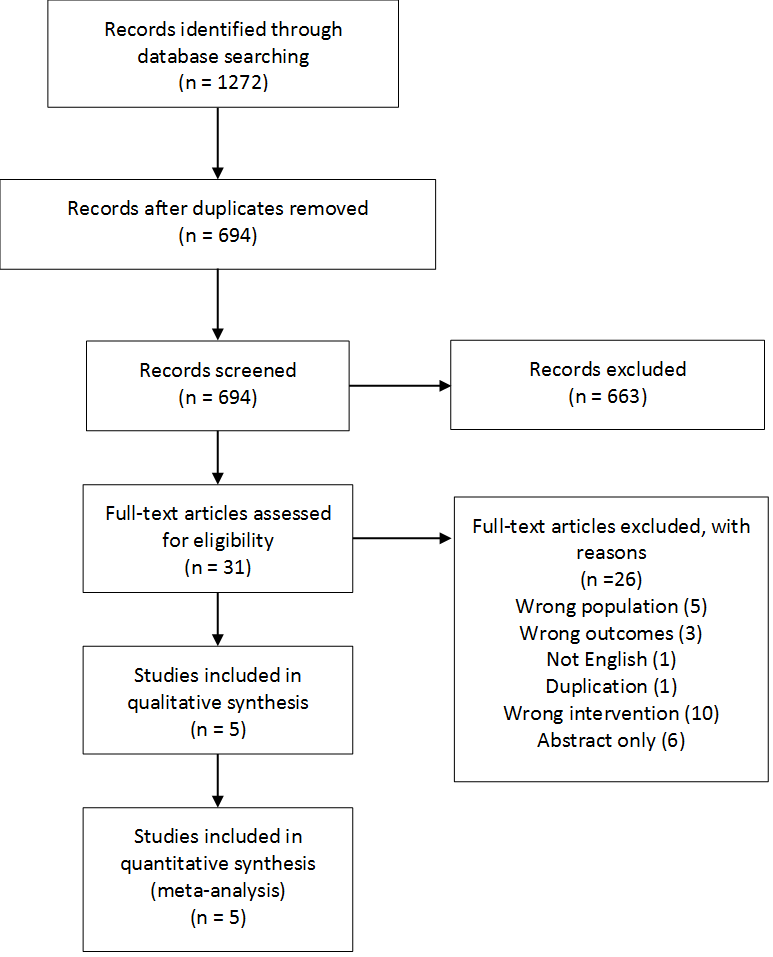
**

**Table S2-S6.**

| ***Summary of Findings Table*** | | | | | | | |
| --- | --- | --- | --- | --- | --- | --- | --- |
| **Author:**  Humaidan 2006 | **Study:**  RCT, single-center, Pilot, open label | | **Title:** Rescue of corpus luteum function with periovulatory  HCG supplementation in IVF/ICSI GnRH antagonist cycles in which ovulation was triggered with a GnRH agonist: a pilot study | | | | |
| **Population** | In/Exclusion criteria:  A total of 45 normogonadotrophic patients submitted to IVF / ICSI cycles in GnRH antagonist protocol with fresh embryo transfer. Trigger applied when at least 3 follicles reached 17mm.  Luteal phase support: Two index arms: HCG 1500 IU at 12h or 35 h post trigger.  All patients received luteal phase support in the form of micronized progesterone vaginally, 90 mg a day (Crinone; Serono Nordic, Copenhagen,  Denmark) and oestradiol 4 mg a day orally (Estrofem; Novo Nordisk, Copenhagen, Denmark) commencing on the day following oocyte retrieval and continuing until the day of the pregnancy test, i.e. day 12 after embryo transfer. | | | | | | |
| **Index group** | GnRHa (Buserelin 0.5 mg) and modified LPS as described above | | | | | | |
| **Reference group** | hCG (Pregnyl 10000 IU) and standard LPS | | | | | | |
| **Critical outcomes** | Index  N=30 | | Reference  N=15 | OR/mean difference | 95%CI | | Comment |
| Live birth | 7/30 | | 8/15 | - | - | | Data also published in Youssef et al. (2014) |
| OHSS moderate/severe  Severe  Moderate | 0/30  -  - | | 0/15  -  - | - | - | |  |
| **Important outcomes** |  | |  |  |  | |  |
| Mean oocytes aspirated | 11.5(6.3) | | 7 (3.5) | 4.54 | 1.66-7.41 | | Mean (SD) |
| Number of M2 oocytes | - | | - | - | - | |  |
| Number of good quality embryos | - | | - | - | - | |  |
| Biochemical pregnancies  Miscarriage | 1/9  1/8 | | 1/9  0/8 |  |  | | Clinical loss/biochemical  live birth loss/clinical |
| Clinical pregnancy/ITT  Clinical pregnancy/ET  Clinical pregnancy/ET | 8/30  8/21  6/12 | | 8/15  8/12  8/12 | 0.32 | 0.09-1.16 | | Only 35h hCG group |
| **Domain** | | **Support for judgement** | | | | **Review authors’ judgement** | |
| **Random sequence generation.** | | Computer generated randomization list | | | | Low risk | |
| **Allocation concealment.** | | Independent nurse, sealed envelopes | | | | Low risk | |
| **Blinding of participants and personnel** | | Open label | | | | High risk | |
| **Blinding of outcome assessment** | | Open label | | | | High risk | |
| **Incomplete outcome data** | | All outcomes reported. No live birth outcome. | | | | Low risk | |
| **Selective reporting** | | Clinical outcomes were reported in a pilot trial, without study report | | | | Unclear risk | |
| **Other sources of bias.** | | Pilot trial | | | | Unclear risk | |

| ***Summary of Findings Table*** | | | | | | | |
| --- | --- | --- | --- | --- | --- | --- | --- |
| **Author:**  Humaidan 2010 | **Study:**  RCT, multicenter, open label | | **Title:** 1,500 IU human chorionic gonadotropin administered  at oocyte retrieval rescues the luteal phase when  gonadotropin-releasing hormone agonist is used for  ovulation induction: a prospective, randomized,  controlled study | | | | |
| **Population** | In/Exclusion criteria:  A total of 302 normogonadotrophic patients submitted to IVF / ICSI cycles in GnRH antagonist protocol with fresh embryo transfer. Trigger applied when at least 2 follicles reached 17mm.  Luteal phase support: In index arm, HCG 1500 IU i.m. at OPU.  For lutealphase support all patients received micronized P (90 mg/d) vaginally (Crinone; Serono Nordic, Copenhagen, Denmark) and E2 (4 mg/d) orally (Estrofem; Novo Nordisk, Copenhagen, Denmark), beginning on the day after oocyte retrieval  and continuing until the day of the pregnancy test (i.e., day 12–14 after ET). | | | | | | |
| **Index group** | GnRHa (Buserelin, Suprefact 0.5 mg) and modified LPS as described above | | | | | | |
| **Reference group** | hCG (Pregnyl 10000 IU s.c.) and standard LPS | | | | | | |
| **Critical outcomes** | Index  N=152 | | Reference  N=150 | OR/ Mean difference | 95%CI | | Comment |
| Live birth/ITT | 36/152 | | 47/150 | 0.68 | 0.41-1.13 | |  |
| OHSS moderate/severe  Severe  Moderate | 0/152  -  - | | 3/150  -1  -2 | 0.14 | 0.01-2.70 | |  |
| **Important outcomes** |  | |  |  |  | |  |
| Mean oocytes aspirated | 8.9 (5.4) | | 9.3(5.0) | -0.40 | (-1.57-0.77) | | Mean (SD) |
| Number of M2 oocytes | 465/546 | | 468/574 | - | - | | M2/ICSI, no mean |
| Number of 2PN embryos | 790/1361 | | 780/1420 | - | - | | IVF/ICSI, no mean |
| Number of good quality embryos | - | | - | - | - | | There is a percentage (30%), no mean |
| Biochemical  Miscarriage | 13/63  14/50 | | 11/66  8/55 |  |  | | Clinical loss/biochemical  live birth loss/clinical |
| Clinical pregnancy/ITT  Clinical pregnancy/ET | 50/152  50/130 | | 55/150  55/138 | 0.85 | 0.53-1.36 | |  |
| Ongoing pregnancy/ITT | 40/152 | | 49/150 | 0.74 | 0.45-1.21 | | Viable pregnancy at 11 weeks’ gestation |
| **Domain** | | **Support for judgement** | | | | **Review authors’ judgement** | |
| **Random sequence generation.** | | Computer generated randomization list | | | | Low risk | |
| **Allocation concealment.** | | Sealed envelopes | | | | Low risk | |
| **Blinding of participants and personnel** | | Open label | | | | High risk | |
| **Blinding of outcome assessment** | | Open label | | | | High risk | |
| **Incomplete outcome data** | | All outcomes reported. | | | | Low risk | |
| **Selective reporting** | | Clinical outcomes were reported as in a pilot trial, without study report | | | | Low risk | |
| **Other sources of bias.** | | None | | | | Low risk | |

| ***Summary of Findings Table*** | | | | | | | |  |  |
| --- | --- | --- | --- | --- | --- | --- | --- | --- | --- |
| **Author:**  Humaidan 2013 | **Study:**  RCT, multicenter, open label | | **Title:** GnRHa trigger and individualized luteal phase hCG support according to ovarian response to stimulation: two prospective randomized controlled multi-centre studies in IVF patients | | | | |  |  |
| **Population** | In/Exclusion criteria:  A total of 384 patients (divided by high risk and low risk of OHSS, cut off 14 follicles on final stimulation day) submitted to IVF / ICSI cycles in GnRH antagonist protocol with fresh embryo transfer. Trigger applied when at least 2 follicles reached 17mm. No patients above 25 follicles.  Luteal phase support: In low risk index arm, patients with ≤14 follicles on the day of triggering received a bolus of 0.5 mg buserelin s.c. followed by a bolus of 1.500 IUhCG s.c. after the oocyte retrieval. Furthermore, patients received an additional bolus of 1.500 IU hCG on the day of oocyte retrieval +5. In high risk, 1.500 IU hCG (Pregnyl) at OPU.  Furthermore, all patients received standard LPS commencing on the day following the oocyte retrieval and continuing until 7 weeks of gestation. | | | | | | |  |  |
| **Index group** | A) High risk GnRHa (Buserelin, Suprefact 0.5 mg sc) and modified LPS  C) Low risk GnRHa (Buserelin, Suprefact 0.5 mg sc) and modified LPS | | | | | | |  |  |
| **Reference group** | B) High risk hCG (Pregnyl 5000 IU s.c.) and standard LPS  D) Low risk hCG (Pregnyl 5000 IU s.c.) and standard LPS | | | | | | |  |  |
| **Critical outcomes** | Index  N=185 | | Reference  N=199 | OR | 95%CI | | Comment | |  |
| Live birth | 49/183 | | 49/199 |  |  | | New data, 2 patients missing in group C | |  |
| OHSS moderate/severe | 2/185 | | 2/199 | 1.08 | 0.15-7.72 | | Only in group C (2/125)  Only in group B (2/58) | |  |
| **Important outcomes** |  | |  |  |  | |  | |  |
| Number of oocytes aspirated | - | | - | - | - | | Presented as medians | |  |
| Number of M2 oocytes | - | | - | - | - | | Presented as medians | |  |
| Number of 2PN embryos |  | |  | - | - | | Presented as medians | |  |
| Number of good quality embryos | - | | - | - | - | | 30%, no mean reported | |  |
| Biochemical  Miscarriage | 8/72  13/62  15/64 | | 5/62  8/57  8/57 |  |  | | Clinical loss/biochemical  live birth loss/clinical excluding missing values  with missing values =miscarriage | |  |
| Clinical pregnancy/ITT  Clinical pregnancy/ET  Clinical pregnancy/group  Clinical pregnancy/group | 64/185  64/162  21/60  43/125 | | 57/199  57/173  17/58  40/141 | 1.32 | 0.86-2.03 | | Overall  Overall  Group A vs Group B  Group C vs Group D | |  |
| Ongoing pregnancy/ITT | 54/185 | | 51/199 | 1.20 | 0.76-1.87 | | Viable pregnancy at 11 weeks’ gestation | |  |
| **Domain** | | | **Support for judgement** | | | | **Review authors’ judgement** | | |
| **Random sequence generation.** | | | Computer generated randomization list | | | | Low risk | | |
| **Allocation concealment.** | | | Sealed envelopes, study nurse | | | | Low risk | | |
| **Blinding of participants and personnel** | | | Open label | | | | High risk | | |
| **Blinding of outcome assessment** | | | Open label | | | | High risk | | |
| **Incomplete outcome data** | | | All outcomes reported. | | | | Low risk | | |
| **Selective reporting** | | | Clinical outcomes were reported recording to trial protocol, however no LBR reported. LBR was collected from the author after publication. | | | | Unclear risk | | |
| **Other sources of bias.** | | | Early discontinuation | | | | Unclear risk | | |

| ***Summary of Findings Table*** | | | | | | | |  |
| --- | --- | --- | --- | --- | --- | --- | --- | --- |
| **Author:**  Papanikolau 2011 | **Study:**  RCT, multicenter, open label, Pilot | | **Title:** A novel method of luteal supplementation with recombinant luteinizing hormone when a gonadotropin-releasing hormone agonist is used instead of human chorionic gonadotropin for ovulation triggering: a randomized prospective proof of concept study | | | | |  |
| **Population** | In/Exclusion criteria:  A total of 35 normogonadotrophic patients submitted to IVF / ICSI cycles in GnRH antagonist protocol with elective single-blastocyst fresh embryo transfer.  Luteal phase support: Standard P luteal support, plus six doses every other day of 300 IU recombinant LH (Luveris, Merck-Serono) starting on the day of oocyte retrieval up to day 10 after oocyte retrieval. | | | | | | |  |
| **Index group** | GnRHa (triptorelin, 0.2mg) and modified LPS as described above | | | | | | |  |
| **Reference group** | rhCG (Ovitrelle 250 mikrog) and standard LPS until week 7 | | | | | | |  |
| **Critical outcomes** | Index  N=18 | | Reference  N=17 | OR/mean difference | 95%CI | | Comment | |
| Live birth/intention to treat  Live birth/ET | 4/18  4/16 | | 4/17  4/15 | 0.93 | 0.19-4.50 | | The patient with monozygotic twins in GnRHa group underwent embryo reduction, personal reasons | |
| OHSS moderate/severe  Severe  Moderate | 0/18  -  - | | 0/17  -  - | - | - | |  | |
| **Important outcomes** |  | |  |  |  | |  | |
| Mean oocytes aspirated | 11.7 (4.3) | | 12.6 (6.6) | -2.1 | -7.23-3.03 | | Mean (SD) | |
| Number of M2 oocytes | - | | - |  |  | | 2PN not M2 | |
| Number of good quality embryos | 43 | | 37 | 0.20 | -1.76-2.16 | | Number of cryopreserved plus embryos transferred | |
| Biochemical  Miscarriage | 1/5  0/4 | | 2/6  0/4 |  |  | | Clinical loss/biochemical  live birth loss/clinical | |
| Clinical pregnancy/ITT  Clinical pregnancy/ET | 4/18  4/16 | | 5/17  5/15 | 0.69 | 0.15-3.15 | | 1 monozygotic twin preg in GnRHa group (thus this is two clinical pregnancies) | |
| Ongoing pregnancy | - | | - | - | - | | Not defined | |
| **Domain** | | **Support for judgement** | | | | **Review authors’ judgement** | | |
| **Random sequence generation.** | | Computer generated randomization list and study nurse | | | | Low risk | | |
| **Allocation concealment.** | | Concealment by study nurse | | | | Low risk | | |
| **Blinding of participants and personnel** | | Blinded until allocation. Probably not following | | | | High risk | | |
| **Blinding of outcome assessment** | | Blinded until allocation. Probably not following | | | | High risk | | |
| **Incomplete outcome data** | | All outcomes reported. | | | | Low risk | | |
| **Selective reporting** | | Clinical outcomes were reported according to protocol | | | | Low risk | | |
| **Other sources of bias.** | | None | | | | Low risk | | |

| ***Summary of Findings Table*** | | | | | | | |
| --- | --- | --- | --- | --- | --- | --- | --- |
| **Author:**  Andersen 2015 | **Study:**  RCT, single-center, open label, Pilot | | **Title:** Daily low-dose hCG stimulation during the luteal phase combined with GnRHa triggered IVF cycles without exogenous progesterone: a proof of concept trial | | | | |
| **Population** | In/Exclusion criteria:  A total of 384 patients submitted to IVF / ICSI cycles in GnRH antagonist protocol with fresh embryo transfer. Trigger applied when at least 3 follicles reached 17mm. No patients above 24 follicles, No Rotterdam PCOS or severe concomitant disease.  Different stimulation with hCG in the GnRHa groups.  Luteal phase support: In index arms, only hCG 125 IU/day from OPU. No exogenous P.  For LPS in reference group, patients received micronized P (90 mg/d) vaginally (Crinone; Serono Nordic, Copenhagen, Denmark) and E2 (4 mg/d) orally (Estrofem; Novo Nordisk, Copenhagen, Denmark), beginning on the day after oocyte retrieval  and continuing until the day of the pregnancy test (i.e., day 12–14 after ET). | | | | | | |
| **Index group** | GnRHa trigger (Buserelin, Suprefact 0.5 mg) and modified LPS as described above | | | | | | |
| **Reference group** | rhCG (Ovitrelle 250mikroG s.c.) and standard LPS | | | | | | |
| **Critical outcomes** | Index  N=61 | | Reference  N=32 | OR/Mean difference | 95%CI | | Comment |
| Live birth | 20/61 | | 11/32 | 0.93 | 0.38-2.30 | |  |
| OHSS moderate/severe  Severe  Moderate | 2/61  -  -2 | | 2/32  -  -2 | 0.51 | 0.07-3.79 | | Criteria according to Humaidan et al. 2013 |
| **Important outcomes** |  | |  |  |  | |  |
| Mean oocytes aspirated | 8.0(0.77) | | 9.3(0.7) | -1.30 | -3.03-0.43 | | Mean(sd) |
| Number of M2 oocytes | - | | - | - | - | | Not reported |
| Number of 2PN embryos | 293 | | 112 | - | - | | Not calculated |
| Number of good quality embryos | 241 | | 90 | 1.15 | 0.10-2.21 | | Grade 0 or 1, day 2-3 embryos |
| Biochemical  Miscarriage | 12/33  1/21 | | 3/15  1/12 |  |  | | Clinical loss/biochemical  live birth loss/clinical |
| Clinical pregnancy/ITT  Clinical pregnancy/ET | 21/61  21/55 | | 12/32  12/29 | 0.88 | 0.36-2.13 | |  |
| Ongoing pregnancy | - | | - | - | - | |  |
| **Domain** | | **Support for judgement** | | | | **Review authors’ judgement** | |
| **Random sequence generation.** | | Computer generated randomization list | | | | Low risk | |
| **Allocation concealment.** | | Sealed envelopes, study nurse concealment | | | | Low risk | |
| **Blinding of participants and personnel** | | Open label | | | | High risk | |
| **Blinding of outcome assessment** | | Open label | | | | High risk | |
| **Incomplete outcome data** | | All outcomes reported. | | | | Low risk | |
| **Selective reporting** | | Clinical outcomes were reported as in study protocol | | | | Low risk | |
| **Other sources of bias.** | | hCG for stimulation | | | | Low risk | |

**3. Sensitivity analysis**

Statistical heterogeneity means that the estimated effects were different across studies, thus adding uncertainty to the results. Therefore, authors of meta-analyses with high heterogeneity should perform sensitivity analyses in an attempt to determine the source of variation across studies. Sensitivity analysis comprises a series of analyses using the dataset to evaluate whether altering any of the assumptions made leads to different results (Thabane et al., 2013).

**Fig. S2 – Sensitivity analysis – excluding study by study – lowest OR**

**
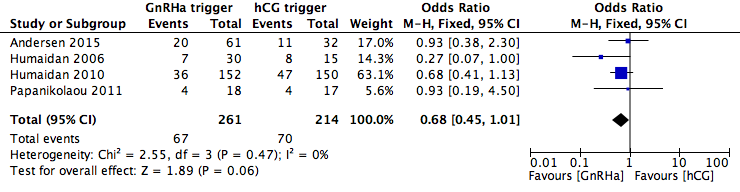
**

**Fig. S3 – Sensitivity analysis – excluding study by study – highest OR**

**
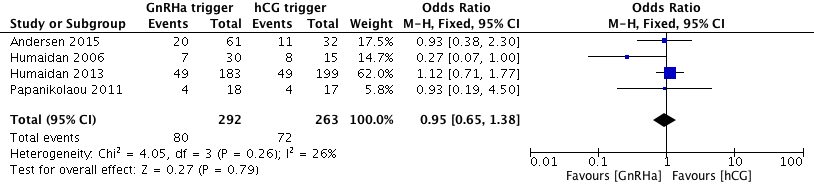
**

**Fig. S4 – Subgroup analysis – Live birth, considering the LLS with hCG bolus at OPU only**


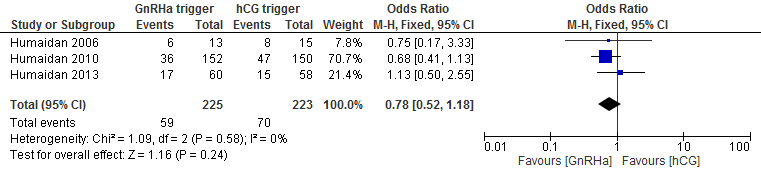


**Table S.7 Sensitivity analysis of outcomes with exclusion of study by study – live birth rate**

| **Live Birth Rate** | | | |
| --- | --- | --- | --- |
| **Included studies** | **Excluded Study** | **Odds Ratio (95% CI)** | ***I^2^*** |
| All | None | 0.84 (0.62, 1.14) | 22% |
| Humaidan et al., 2006  Humaidan et al., 2010  Humaidan et al., 2013  Papanikolaou et al., 2011 | Andersen et al., 2015 | 0.83 (0.60, 1.15) | 41% |
| Andersen et al., 2015  Humaidan et al., 2010  Humaidan et al., 2013  Papanikolaou et al., 2011 | Humaidan et al., 2006 | 0.90 (0.66, 1.23) | 0% |
| Andersen et al., 2015  Humaidan et al., 2006  Humaidan et al., 2013  Papanikolaou et al., 2011 | Humaidan et al., 2010 | 0.95 (0.65, 1.38) | 26% |
| Andersen et al., 2015  Humaidan et al., 2006  Humaidan et al., 2010  Papanikolaou et al., 2011 | Humaidan et al., 2013 | 0.68 (0.45, 1.01) | 0% |
| Andersen et al., 2015  Humaidan et al., 2006  Humaidan et al., 2010  Humaidan et al., 2013  Papanikolaou et al., 2011 | Papanikolaou et al., 2011 | 0.84 (0.62, 1.14) | 41% |

**Table S.8 Sensitivity analysis of outcomes with exclusion of study by study – OHSS**

| **OHSS** | | | |
| --- | --- | --- | --- |
| **Included studies** | **Excluded Study** | **Odds Ratio (95% CI)** | ***I^2^*** |
| All | None | 0.48 (0.15, 1.60) | 0% |
| Humaidan et al., 2006  Humaidan et al., 2010  Humaidan et al., 2013  Papanikolaou et al., 2011 | Andersen et al., 2015 | 0.47 (0.11, 2.09) | 25% |
| Andersen et al., 2015  Humaidan et al., 2010  Humaidan et al., 2013  Papanikolaou et al., 2011 | Humaidan et al., 2006 | 0.48 (0.15, 1.60) | 0% |
| Andersen et al., 2015  Humaidan et al., 2006  Humaidan et al., 2013  Papanikolaou et al., 2011 | Humaidan et al., 2010 | 0.75 (0.18, 3.07) | 0% |
| Andersen et al., 2015  Humaidan et al., 2006  Humaidan et al., 2010  Papanikolaou et al., 2011 | Humaidan et al., 2013 | 0.29 (0.06, 1.43) | 0% |
| Andersen et al., 2015  Humaidan et al., 2006  Humaidan et al., 2010  Humaidan et al., 2013  Papanikolaou et al., 2011 | Papanikolaou et al., 2011 | 0.48 (0.15, 1.60) | 0% |

**Table S.9 Sensitivity analysis of outcomes with exclusion of study by study – clinical pregnancy rate**

| **Clinical Pregnancy Rate** | | | |
| --- | --- | --- | --- |
| **Included studies** | **Excluded Study** | **Odds Ratio (95% CI)** | ***I^2^*** |
| All | None | 0.99 (0.74, 1.32) | 22% |
| Humaidan et al., 2006  Humaidan et al., 2010  Humaidan et al., 2013  Papanikolaou et al., 2011 | Andersen et al., 2015 | 1.00 (0.74, 1.36) | 41% |
| Andersen et al., 2015  Humaidan et al., 2010  Humaidan et al., 2013  Papanikolaou et al., 2011 | Humaidan et al., 2006 | 1.05 (0.78, 1.41) | 0% |
| Andersen et al., 2015  Humaidan et al., 2006  Humaidan et al., 2013  Papanikolaou et al., 2011 | Humaidan et al., 2010 | 1.08 (0.75, 1.55) | 33% |
| Andersen et al., 2015  Humaidan et al., 2006  Humaidan et al., 2010  Papanikolaou et al., 2011 | Humaidan et al., 2013 | 0.79 (0.53, 1.15) | 0% |
| Andersen et al., 2015  Humaidan et al., 2006  Humaidan et al., 2010  Humaidan et al., 2013  Papanikolaou et al., 2011 | Papanikolaou et al., 2011 | 0.99 (0.74, 1.32) | 41% |

**Table S.10 Sensitivity analysis of outcomes with exclusion of study by study – miscarriage rate**

| **Miscarriage Rate** | | | |
| --- | --- | --- | --- |
| **Included studies** | **Excluded Study** | **Odds Ratio (95% CI)** | ***I^2^*** |
| All | None | 1.85 (0.97, 3.54) | 0% |
| Humaidan et al., 2006  Humaidan et al., 2010  Humaidan et al., 2013  Papanikolaou et al., 2011 | Andersen et al., 2015 | 1.97 (1.01, 3.85) | 0% |
| Andersen et al., 2015  Humaidan et al., 2010  Humaidan et al., 2013  Papanikolaou et al., 2011 | Humaidan et al., 2006 | 1.80 (0.93, 3.49) | 0% |
| Andersen et al., 2015  Humaidan et al., 2006  Humaidan et al., 2013  Papanikolaou et al., 2011 | Humaidan et al., 2010 | 1.56 (0.65, 3.73) | 0% |
| Andersen et al., 2015  Humaidan et al., 2006  Humaidan et al., 2010  Papanikolaou et al., 2011 | Humaidan et al., 2013 | 2.05 (0.85, 4.95) | 0% |
| Andersen et al., 2015  Humaidan et al., 2006  Humaidan et al., 2010  Humaidan et al., 2013  Papanikolaou et al., 2011 | Papanikolaou et al., 2011 | 1.85 (0.97, 3.54) | 0% |

**Table S.11 Sensitivity analysis of outcomes with exclusion of study by study – oocytes retrieved**

| **Oocytes Retrieved** | | | |
| --- | --- | --- | --- |
| **Included studies** | **Excluded Study** | **Mean Difference (95% CI)** | ***I^2^*** |
| All | None | 0.25 (-2.03, 2.53) | 76%% |
| Humaidan et al., 2006  Humaidan et al., 2010  Papanikolaou et al., 2011 | Andersen et al., 2015 | 0.90 (-2.80, 4.60) | 81% |
| Andersen et al., 2015  Humaidan et al., 2010  Papanikolaou et al., 2011 | Humaidan et al., 2006 | -0.73 (-1.69, 0.22) | 0% |
| Andersen et al., 2015  Humaidan et al., 2006  Papanikolaou et al., 2011 | Humaidan et al., 2010 | 0.53 (-3.76, 4.83) | 84% |
| Andersen et al., 2015  Humaidan et al., 2006  Humaidan et al., 2010  Papanikolaou et al., 2011 | Papanikolaou et al., 2011 | 0.63 (-1.94, 3.21) | 83% |

**4. Publication bias**

Absence of obvious publication bias is suggested when most of the data appeared at the top of a funnel plot and were distributed roughly symmetrically (Egger et al. 1997). That is, we would expect studies to be dispersed equally on either side of the overall effect. As seen in **Supplementary Figure 4**, the funnel plots are actually symmetric for all outcome measures with an overall similar number of studies falling toward the left and right of the mean effect.

Funnel plots corresponding to the meta-analyses of primary outcomes.

**LBR**

***
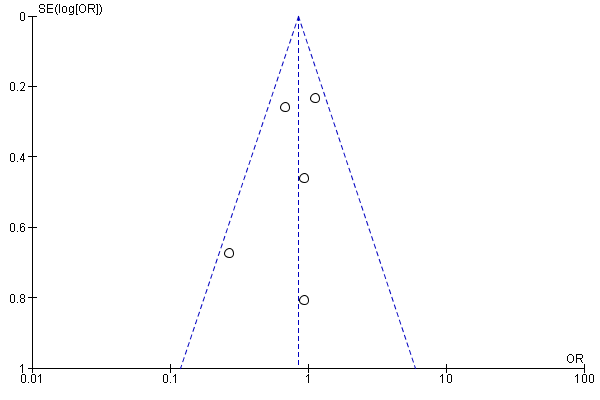
***

**OHSS**

***
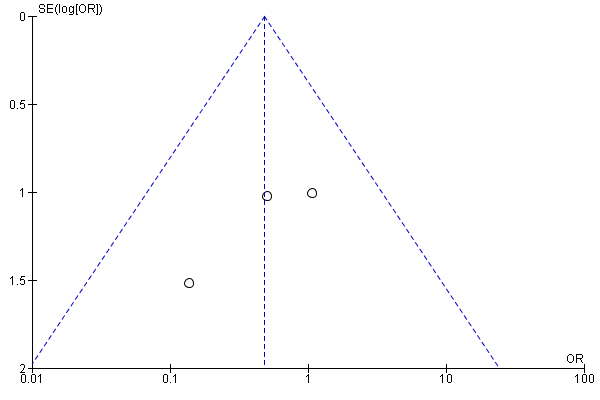
***

***References***

Thabane L, Mbuagbaw L, Zhang S, Samaan Z, Marcucci M, Ye C, Thabane M, Giangregorio L, Dennis B, Kosa D, Borg Debono V, Dillenburg R, Fruci V, Bawor M, Lee J, Wells G, Goldsmith CH. A tutorial on sensitivity analyses in clinical trials: the what, why, when and how. BMC Med Res Methodol 2013;13:92.

1. **Literature search:**

Database: Embase <1974 to 2016 December 13>

Search Strategy:

--------------------------------------------------------------------------------

1 gonadorelin derivative/ or buserelin/ or buserelin acetate/ or deslorelin/ or folligen/ or gonadorelin/ or gonadorelin acetate/ or gonadorelin agonist/ or goserelin/ or histrelin/ or leuprorelin/ or lutrelin/ or nafarelin/ or nafarelin acetate/ or ovurelin/ or triptorelin/ (64864)

2 gonadorelin$.tw. (324)

3 gonadotropin-releasing hormone$.tw. (13710)

4 (buserelin or Suprefact).tw. (2273)

5 (goserelin or Zoladex).tw. (2908)

6 (leuprolide or lupron).tw. (3821)

7 (nafarelin or Synarel).tw. (603)

8 (histrelin or Supprelin).tw. (131)

9 (deslorelin or Suprelorin or Ovuplant).tw. (257)

10 triptorelin$.tw. (1051)

11 gonadotropin-releasing hormone agonist$.tw. (2279)

12 gonadotrophin releasing hormone agonist$.tw. (556)

13 GnRH agonist$.tw. (5604)

14 GnRH a.tw. (1221)

15 GnRHa.tw. (1851)

16 or/1-15 (67566)

17 trigger$.tw. (268847)

18 (oocyte adj5 matur$).tw. (9153)

19 (ovulat$ adj2 induc$).tw. (9181)

20 or/17-19 (285673)

21 16 and 20 (4075)

22 randomized controlled trial/ (467113)

23 exp randomization/ (83985)

24 single blind procedure/ (27944)

25 double blind procedure/ (138777)

26 placebo/ (328280)

27 Randomi?ed controlled trial$.tw. (150938)

28 Rct.tw. (22645)

29 random allocation.tw. (1646)

30 randomly allocated.tw. (26811)

31 allocated randomly.tw. (2212)

32 (allocated adj2 random).tw. (854)

33 Single blind$.tw. (18831)

34 Double blind$.tw. (175600)

35 ((treble or triple) adj blind$).tw. (662)

36 placebo$.tw. (250013)

37 case study/ (93638)

38 abstract report/ or letter/ (1009856)

39 exp clinical trial/ (1284340)

40 22 or 23 or 24 or 25 or 26 or 27 or 28 or 29 or 30 or 31 or 32 or 33 or 34 or 35 or 36 or 39 (1597526)

41 case report/ (2173964)

42 37 or 38 or 41 (3072441)

43 40 not 42 (1535568)

44 21 and 43 (741)

45 human/ (18133618)

46 exp animal/ (22763082)

47 46 not 45 (4629464)

48 44 not 47 (651)

49 limit 48 to embase (217)

50 limit 49 to yr="2000 -Current" (208)

PubMed 14122016:

((((((((((((((((((((("Gonadotropin-Releasing Hormone"[Mesh]) OR gonadorelin*[Text Word]) OR gonadotropin-releasing hormone*[Text Word]) OR ((buserelin[Text Word] OR Suprefact[Text Word]))) OR ((goserelin[Text Word] OR Zoladex[Text Word]))) OR ((leuprolide[Text Word] OR lupron[Text Word]))) OR ((nafarelin[Text Word] OR Synarel[Text Word]))) OR ((histrelin[Text Word] OR Supprelin[Text Word]))) OR ((deslorelin[Text Word] OR Suprelorin[Text Word] OR Ovuplant[Text Word]))) OR triptorelin*[Text Word]) OR gonadotrophin releasing hormone*) OR GnRH agonist*[Text Word]) OR GnRH a[Text Word]) OR GnRHa[Text Word])) AND (((trigger*[Text Word]) OR ovulation induct*[Text Word]) OR induction of ovulat*[Text Word]))) AND ((((((((((((((((("Clinical Trial" [Publication Type]) OR "Randomized Controlled Trial" [Publication Type]) OR (("Single-Blind Method"[Mesh]) OR "Double-Blind Method"[Mesh])) OR "Placebos"[Mesh]) OR Randomised controlled trial*[Text Word]) OR Randomized controlled trial*[Text Word]) OR Rct[Text Word]) OR allocated random*[Text Word]) OR random allocat*[Text Word]) OR randomly allocat*[Text Word]) OR Single blind*[Text Word]) OR Double blind*[Text Word]) OR treble blind*[Text Word]) OR triple blind[Text Word]) OR placebo*[Text Word])) NOT (("Case Reports" [Publication Type]) OR "Letter" [Publication Type])))) NOT (("Animals"[Mesh]) NOT "Humans"[Mesh])) AND ( "2000/01/01"[PDat] : "3000/12/31"[PDat] ))

**6. PRISMA checklist**

| **Section/topic** | **#** | **Checklist item** | **Reported on page #** |
| --- | --- | --- | --- |
| **TITLE** | | |  |
| Title | 1 | Identify the report as a systematic review, meta-analysis, or both. | 1 |
| **ABSTRACT** | | |  |
| Structured summary | 2 | Provide a structured summary including, as applicable: background; objectives; data sources; study eligibility criteria, participants, and interventions; study appraisal and synthesis methods; results; limitations; conclusions and implications of key findings; systematic review registration number. | 2 |
| **INTRODUCTION** | | |  |
| Rationale | 3 | Describe the rationale for the review in the context of what is already known. | 4-5 |
| Objectives | 4 | Provide an explicit statement of questions being addressed with reference to participants, interventions, comparisons, outcomes, and study design (PICOS). | 5-6 and Table S.1 |
| **METHODS** | | |  |
| Protocol and registration | 5 | Indicate if a review protocol exists, if and where it can be accessed (e.g., Web address), and, if available, provide registration information including registration number. | 4 |
| Eligibility criteria | 6 | Specify study characteristics (e.g., PICOS, length of follow-up) and report characteristics (e.g., years considered, language, publication status) used as criteria for eligibility, giving rationale. | 5-7 and Supplemental Table 1 |
| Information sources | 7 | Describe all information sources (e.g., databases with dates of coverage, contact with study authors to identify additional studies) in the search and date last searched. | 5 |
| Search | 8 | Present full electronic search strategy for at least one database, including any limits used, such that it could be repeated. | Supplementary material |
| Study selection | 9 | State the process for selecting studies (i.e., screening, eligibility, included in systematic review, and, if applicable, included in the meta-analysis). | 5-6 |
| Data collection process | 10 | Describe method of data extraction from reports (e.g., piloted forms, independently, in duplicate) and any processes for obtaining and confirming data from investigators. | 5-6 |
| Data items | 11 | List and define all variables for which data were sought (e.g., PICOS, funding sources) and any assumptions and simplifications made. | 5-7 and Supplemental Table 1; |
| Risk of bias in individual studies | 12 | Describe methods used for assessing risk of bias of individual studies (including specification of whether this was done at the study or outcome level), and how this information is to be used in any data synthesis. | 6 and supplementary tables 2-6 |
| Summary measures | 13 | State the principal summary measures (e.g., risk ratio, difference in means). | 6-7 |
| Synthesis of results | 14 | Describe the methods of handling data and combining results of studies, if done, including measures of consistency (e.g., I^2^) for each meta-analysis. | 7 |

Page 1 of 2

| **Section/topic** | **#** | **Checklist item** | **Reported on page #** |
| --- | --- | --- | --- |
| Risk of bias across studies | 15 | Specify any assessment of risk of bias that may affect the cumulative evidence (e.g., publication bias, selective reporting within studies). | Table 1 |
| Additional analyses | 16 | Describe methods of additional analyses (e.g., sensitivity or subgroup analyses, meta-regression), if done, indicating which were pre-specified. | 7 |
| **RESULTS** | | |  |
| Study selection | 17 | Give numbers of studies screened, assessed for eligibility, and included in the review, with reasons for exclusions at each stage, ideally with a flow diagram. | Supplemental figure 1 |
| Study characteristics | 18 | For each study, present characteristics for which data were extracted (e.g., study size, PICOS, follow-up period) and provide the citations. | Table 1 |
| Risk of bias within studies | 19 | Present data on risk of bias of each study and, if available, any outcome level assessment (see item 12). | Table 1 and supplementary tables 2-6 |
| Results of individual studies | 20 | For all outcomes considered (benefits or harms), present, for each study: (a) simple summary data for each intervention group (b) effect estimates and confidence intervals, ideally with a forest plot. | Table 1 |
| Synthesis of results | 21 | Present results of each meta-analysis done, including confidence intervals and measures of consistency. | 7-9 |
| Risk of bias across studies | 22 | Present results of any assessment of risk of bias across studies (see Item 15). | Table 1 |
| Additional analysis | 23 | Give results of additional analyses, if done (e.g., sensitivity or subgroup analyses, meta-regression [see Item 16]). | 7-8 and Supplemental figures 2-6 and suppl. tables 7-10- |
| **DISCUSSION** | | |  |
| Summary of evidence | 24 | Summarize the main findings including the strength of evidence for each main outcome; consider their relevance to key groups (e.g., healthcare providers, users, and policy makers). | Table 1 |
| Limitations | 25 | Discuss limitations at study and outcome level (e.g., risk of bias), and at review-level (e.g., incomplete retrieval of identified research, reporting bias). | 12-13 |
| Conclusions | 26 | Provide a general interpretation of the results in the context of other evidence, and implications for future research. | 10-12 |
| **FUNDING** | | |  |
| Funding | 27 | Describe sources of funding for the systematic review and other support (e.g., supply of data); role of funders for the systematic review. | 14 |
